# A Rare Case of Obliterated Femoral Canal in Atypical Femoral Shaft Fracture Related to Bisphosphonate Therapy

**DOI:** 10.7759/cureus.45441

**Published:** 2023-09-18

**Authors:** Riko Febrian Kunta Adjie, Mohd Yazid Bajuri, Nik Alif Nik Abdullah, Juzaily F Leong

**Affiliations:** 1 Orthopaedics and Traumatology, Faculty of Medicine, Universiti Kebangsaan Malaysia, Kuala Lumpur, MYS

**Keywords:** intramedullary nailing (imn), atypical femur fracture, geriatric, bisphosphonate, osteoporosis

## Abstract

Bisphosphonates have been accepted as the first-line treatment for postmenopausal osteoporosis. Atypical femoral shaft fracture is one of the side effects of long-term bisphosphonate therapy. The mainstay treatment of this atypical fracture is bisphosphonates cessation and stabilization with internal fixation. We are reporting a rare case of a blocked intramedullary femoral canal found during surgery of an 85-year-old Indian lady with an atypical femoral shaft fracture related to her five-year alendronate therapy. We found difficulty in passing the guidewire through the fracture site during the closed method, which renders open reduction to manage the obliterated intramedullary canal. The importance of changing decisions intraoperatively should be highlighted to avoid further complications. Fracture union is achieved during our follow-up in the clinic.

## Introduction

Osteoporosis is characterized by reduced bone mass leading to fragile bones prone to fractures. Bisphosphonates are commonly prescribed as the first-line treatment, especially for elderly individuals, to mitigate the risk of fragility fractures. These antiresorptive medications hinder excessive bone turnover. Nevertheless, their extended usage can result in rare, atypical femoral fractures [1,2].

Atypical femoral fractures are defined as insufficiency fractures that occur in the subtrochanteric or diaphyseal area of the femur. They may develop in individuals with underlying issues related to metabolic bone disease. However, they are most commonly associated with the prolonged use of bisphosphonate medications, recognized as a significant side effect. Usage exceeding three years escalates the likelihood of such fractures. Although these atypical fractures are infrequent, their occurrence has been on the rise, with the rate increasing from 1.8 to 133 cases per 100,000 individuals per year for bisphosphonate usage under two years and over two years, respectively. It is widely acknowledged that bisphosphonates modify the activity of osteoclasts, hence bone remodeling capacity that leads to the accumulation of microscopic damage and the development of stress fractures [3]. In some rare occurrences, a change in the femur's intramedullary canal structure can obstruct intramedullary nailing, a situation rarely encountered [4-6]. In this article, we present a case involving a blocked intramedullary canal in the context of an atypical femoral fracture, detailing its management.

## Case presentation

An 85-year-old Indian woman presented with a proximal femur fracture at our orthopedic clinic. She had complained of right hip pain for the past week and was unable to ambulate, necessitating the use of a wheelchair. She reported that her right leg "gave way" but denied any fall or trauma. She had been on oral alendronate for five years when she was admitted to the hospital. Otherwise, she had no night pain, fever, significant loss of weight, or any other constitutional symptoms. Her underlying medical problems were diabetes and hypertension. No history of malignancy or metabolic bone disease previously was noted.

**Figure 1:**
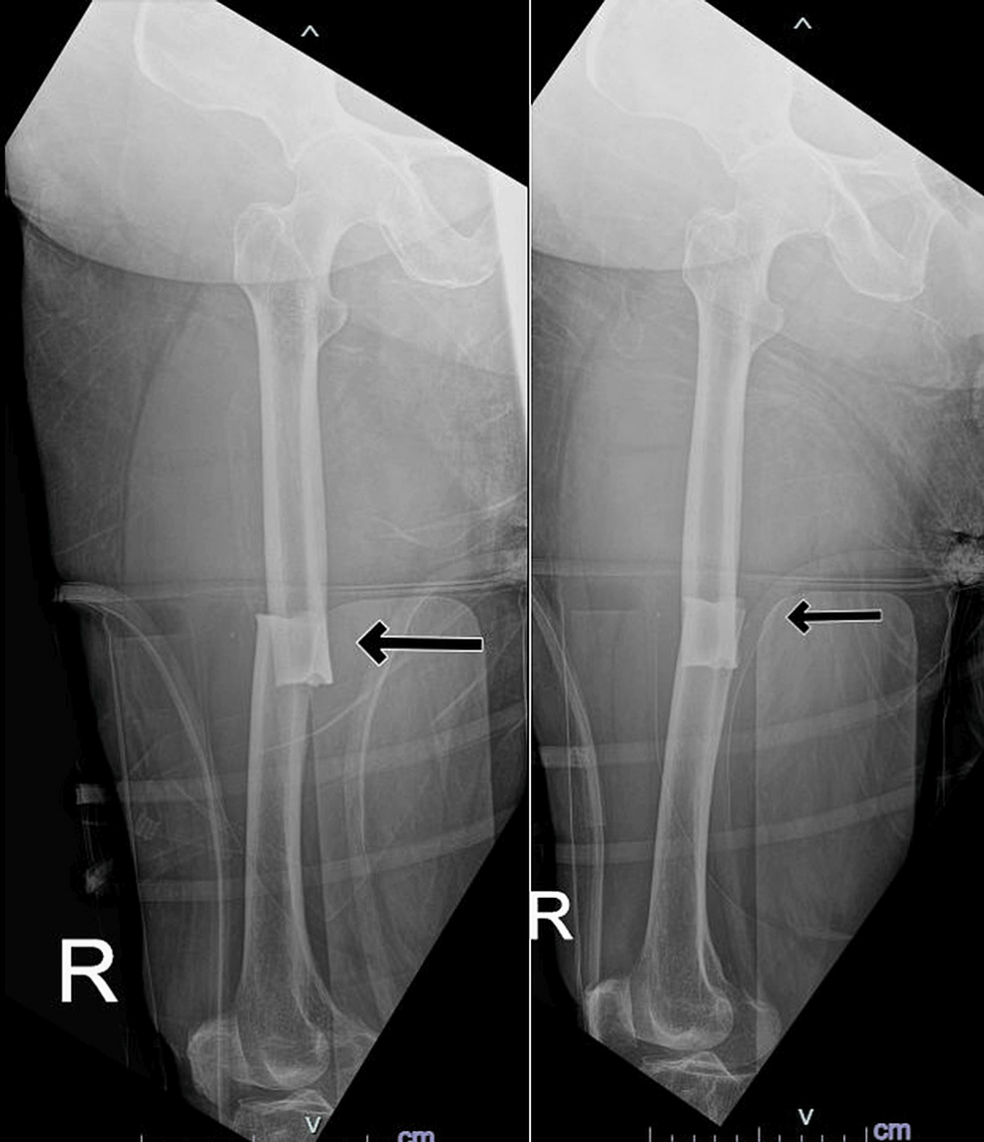
Preoperative radiograph of the patient showing atypical femoral fracture.

Upon examination, her right thigh was tender, which limited her movement. But it did not show obvious deformity. The skin was intact with no bruises. The distal neurovascular function was intact. On plain radiographs, it showed a transverse midshaft femur fracture with cortical thickening in both lateral-medial cortex (Figure 1). Her full blood count, electrolyte levels, calcium blood levels, and renal function were all within the normal range.

**Figure 2:**
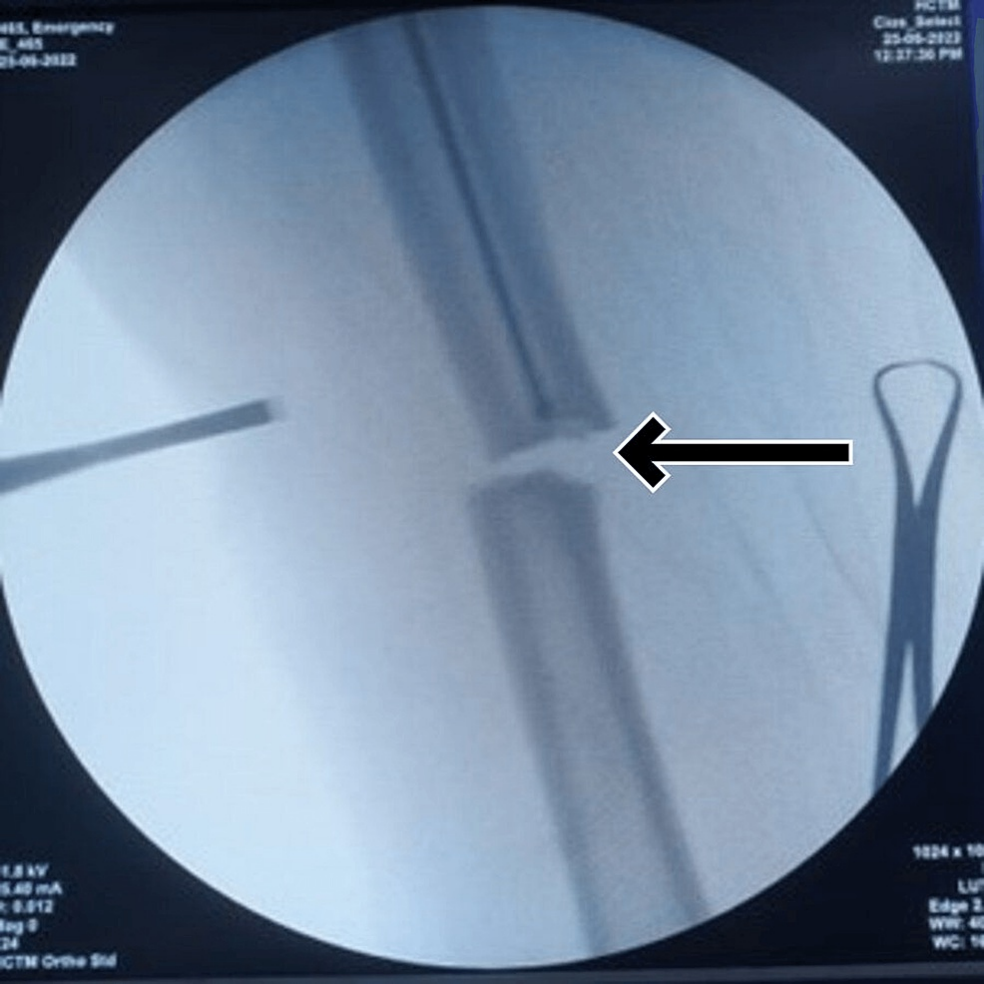
Obliterated intramedullary canal found during guidewire insertion.

**Figure 3:**
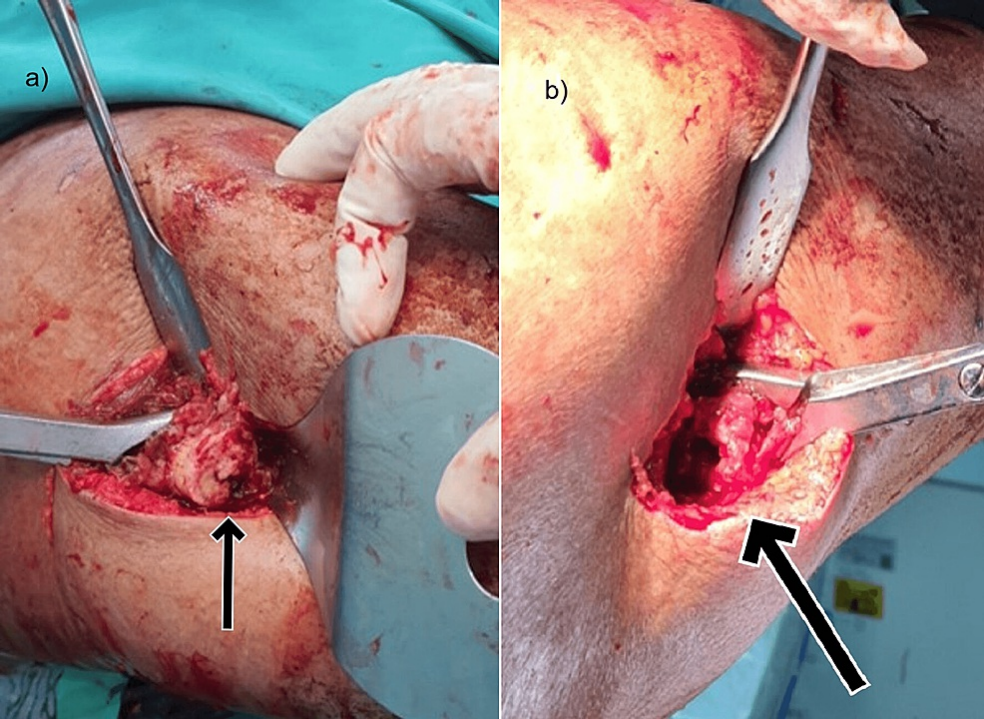
(a) The proximal part of the femur fragment was obliterated and needed to be manually drilled. (b) The distal part of the fracture site was not blocked with bony-hard material.

Operative fixation using an intramedullary nail was performed. Initially, we planned for a close reduction of the fracture. However, intraoperatively, we were unable to pass the guidewire through the fracture site. Subsequently, we observed on the image intensifier that the canal was blocked. After attempting several times to clear the blockage with mallet tapping, we still could not pass the guidewire through (Figure 2). At that point, we decided not to attempt closed reaming and instead opted for open reduction. During the procedure, we discovered that the proximal part of the canal was obliterated (Figure 3). A hard-dense bone material was obliterating the intramedullary cavity proximal to the fracture site. The proximal fragment had to be drilled to re-create the intramedullary canal, and the subsequent guidewire was able to pass through greater trochanter tip insertion and continued with alternate reaming.

**Figure 4:**
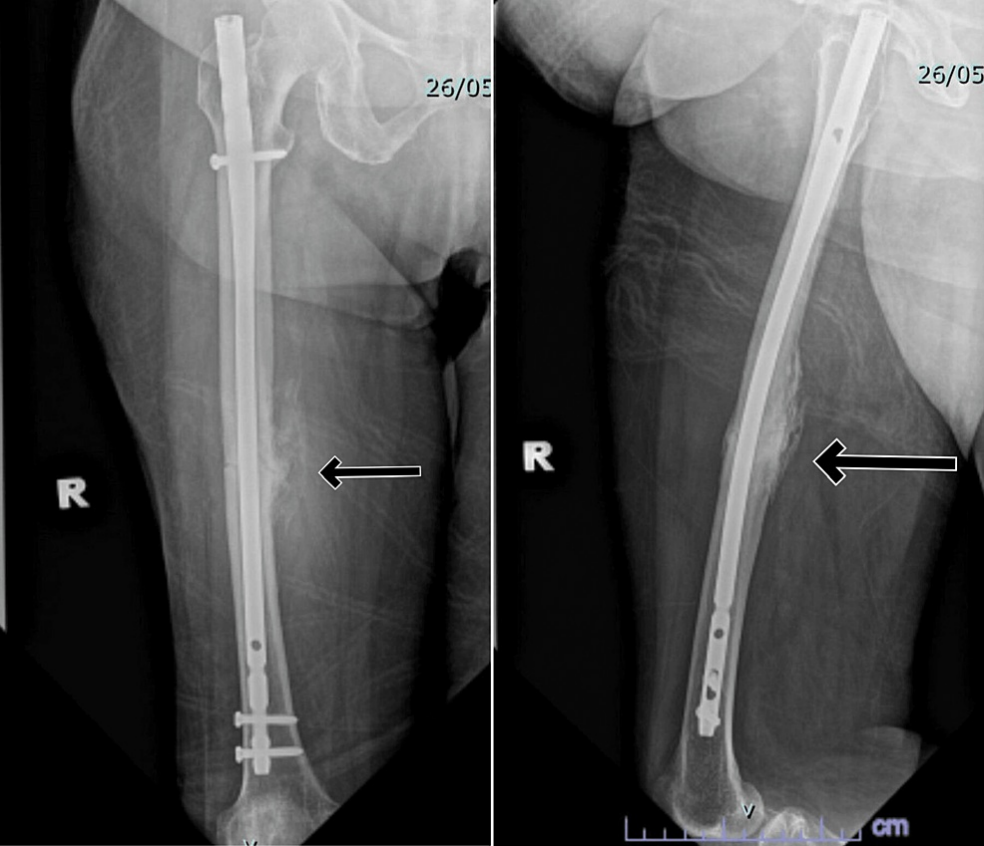
Anteroposterior and lateral view plain radiographs showing the union of the right femur nine months postoperatively.

Postoperatively, the patient's recovery was uneventful, and she was subsequently discharged. Her alendronate therapy was discontinued, but she continued to receive vitamin D and calcium supplements. We did not start any anabolic agent for this case. She was continuing with wheelchair ambulation initially. After the fracture site showed callus formation and her pain had improved, we allowed her to gradual weight-bearing. At the nine-month follow-up, the patient has already demonstrated clinical and radiological union (Figure 4).

## Discussion

In 2005, Odvina et al. [1] first reported femoral fractures related to bisphosphonate use, and subsequently, several more cases of atypical femoral fractures related to bisphosphonate use were identified. These fractures typically exhibit transverse patterns and occur at the tensile part of the femur bone. Atypical features are characterized by lateral cortical breaking, fracture line located between the distal lesser trochanter and the proximal edge of the supracondylar region, and transverse or oblique pattern without any comminution that is accompanied by increasing pain over the thigh area, related to a history minimal trauma. Only 1.1% of femoral fractures are accounted for atypical causes [3]. Although it is rare, the increment of this kind of atypical fracture diagnosis has led to more reporting cases of complications and technical difficulty during the intraoperative period [4-6].

In our case, the patient presented with a fracture with a history of prolonged bisphosphonate use for more than three years, although there was no history of trivial trauma. The fracture line was transverse in the middle third of the shaft femur. Following the diagnosis of an atypical femoral fracture, bisphosphonate therapy has been discontinued and fixation with intramedullary nailing is taken [2,3]. The standard treatment for atypical femoral fractures is stabilization with intramedullary nail fixation [3]. Preoperatively, due to bisphosphonate-related cause fractures, we anticipate that the cortex appears to be thickened and predisposes to a higher risk of intraoperative comminution [5]. The reduction method can be done either closely or openly. Initially, we prefer to do a closed reduction to avoid disturbing the fracture zone. However, due to the blocked intramedullary canal during guidewire insertion, we proceeded with open reduction. Although the latter technique is related to a higher risk of delayed union and infection rate, it was deemed safer to manually ream the obstructed canal.

We found three cases [4-6] of obliterated intramedullary canals during intramedullary nailing for atypical femoral fractures. Such an abnormal morphology is scarcely documented. All cases required open reduction and manual reaming to clear the canal blockage. Closed reaming has not been reported for these cases. We speculated that closed reaming might lead to drill bit breakage or excessive heat generation, resulting in bone necrosis or further comminution, jeopardizing fixation, or impeding the healing process. Once the canal was restored, we employed alternating intramedullary reaming techniques. Patience and caution were imperative when drilling and reaming the brittle osteopetrosis bone with a blocked marrow cavity [4]. Nonetheless, our case emphasizes the importance of anticipating intraoperative challenges when managing atypical femoral fractures.

The obliterated intramedullary canal portion likely results from inhibited bone remodeling due to prolonged alendronate use. Alendronate should be discontinued upon diagnosis. In select cases, teriparatide, an anabolic treatment, may aid healing of atypical femoral fractures [3]. We did not introduce any anabolic agents to enhance healing. While some cases might be complicated with non-union [4], our case achieved union. Fracture consolidation occurred nine months post-intramedullary nailing. The decision to adjunct an anabolic agent should be made on a case-by-case basis.

## Conclusions

With the increased usage of bisphosphonates among geriatric populations, atypical femoral fracture incidence might increase in the future. A rare occurrence of a blocked intramedullary canal can complicate intraoperatively. Awareness of the challenges in intraoperative nailing of atypical femoral fractures is crucial. Open reduction with in situ drilling becomes necessary, instead of closed reduction and continuous reaming, which could cause further complications. We showed that open reduction and direct drilling into the intramedullary canal from the fracture site can still achieve the union of this atypical fracture.
